# Influence of local habitat on the physiological responses of large benthic foraminifera to temperature and nutrient stress

**DOI:** 10.1038/srep21936

**Published:** 2016-02-23

**Authors:** Martina Prazeres, Sven Uthicke, John M. Pandolfi

**Affiliations:** 1 Australian Research Council (ARC) Centre of Excellence for Coral Reef Studies and School of Biological Sciences, The University of Queensland, Brisbane, Queensland, 4072 Australia; 2 Australian Institute of Marine Science, PMB No 3, Townsville, Queensland, 4810 Australia

## Abstract

Large benthic foraminifera (LBF) are important for reef sediment formation, but sensitive to elevated temperature and nutrients. However, it is possible that conspecific foraminifera living in different reef sites present divergent response to environmental shifts. We investigated how populations of *Amphistegina lobifera* from reef sites located along a temperature and nutrient gradient of the northern Great Barrier Reef respond and acclimate to elevated temperature and nitrate under lab-controlled conditions. Generalized linear mixed models showed that interaction between reef sites and temperature or nitrate conditions had a significant effect on survivorship, bleaching frequency and growth rates of *A. lobifera*. Further physiological analyses of antioxidant capacity and Ca-ATPase activity showed that populations collected from the inner-shelf sites (highest nutrient levels, largest temperature variation) were consistently able to acclimate to both parameters after 30 days. In contrast, foraminifera collected from the reef sites located in the mid- and outer-shelfs were significantly more sensitive to elevated temperatures and nitrate. Our results highlight the importance of local habitat in shaping the tolerance of LBF to changing environmental conditions; populations that live in stable environments are more sensitive to elevated temperature and nitrate, even within their fundamental tolerance range, than those that experience fluctuating conditions.

Worldwide, coral reefs are rapidly declining due to deteriorating environmental conditions driven by climate change^1^ and local impacts such as overfishing and terrestrial runoff^2^. As sea-surface temperatures (SST) increase under climate change, research investigating thermo-tolerance of reef organisms and identifying resistant/resilient populations has become increasingly important to identify holobiont systems that will, or could, have the ability to adapt and acclimate to rapidly changing environments^3^,^4^,^5^. The environmental degradation of coral reefs, coral bleaching and ocean warming have all kindled general interest in the adaptive value and stability of algal- invertebrate symbioses in these environments^6^, since most crucial reef calcifiers and reef-building organisms rely on the symbiosis with algae to survive^7^,^8^.

Photosymbiont-bearing large benthic foraminifera (LBF) are single-celled protists that build a calcium carbonate (CaCO_3_) shell, and harbor algae as symbionts, providing their host with energy for growth and calcification^9^. They are restricted to a narrow set of environmental conditions, such as the relatively clear nutrient-poor waters of tropical and warm-temperate seas^6^,^9^,^10^. LBFs represent a unique and important group of organisms that are vital to coral reef ecosystems^11^. They play a crucial role in carbonate cycling in coral reef environments, contributing up to 80% of the global foraminiferal reef carbonate production^12^. Two of the main factors influencing the distribution of LBF include temperature and food availability, such as nutrients^13^.

*Amphistegina* is the most common and abundant LBF genus found in coral reefs worldwide^9^, and hosts diatoms as symbionts^6^. Exposure to temperatures above a threshold value, which is often a few degrees higher than the local summer maxima, can negatively affect photosynthesis, growth rates and increase bleaching frequency^14^,^15^,^16^. Talge and Hallock^17^ showed that, in *A. gibbosa*, bleaching can be triggered by exposure to temperature above their normal thermal range, but without mortality. Indeed, Schmidt *et al.*^15^ reported bleaching without mortality of *A. radiata* individuals collected from the Whitsundays region on the central Great Barrier Reef (GBR) exposed to 31 °C (3 °C above their summer maximum) for 30 days.

Terrestrial run-off and upwelling can also have negative effects on calcifying organisms that host algae as symbionts. Input of nitrate and phosphates stimulates growth of plankton, which reduces water transparency. This limits depth ranges of photosymbiont organisms, such as corals and calcareous algae, reducing carbonate production^18^,^19^. Additionally, elevated concentrations of dissolved nutrient are often associated with reduced growth in LBF^20^,^21^, as the increased nutrient availability releases foraminiferal symbionts from nutrient limitation^20^. Therefore, temperatures and nutrient concentrations above their natural range could potentially reduce densities of LBF on reefs affected by fluctuations and abnormal peaks of these two parameters.

In LBFs, the host relies on the symbiotic algae for growth and calcification, and to meet its metabolic demands^8^,^22^. As such, the physiological performance of the symbionts can greatly affect the overall health of the host, as well as the holobiont as a whole^11^. As coral reef ecosystems face unprecedented changes, identifying physiological traits that contribute to enhancing performance of the response of foraminiferal populations to different environmental conditions can provide simple and essential information on how populations of the same species are able to live in various habitats.

Here, we quantified how populations of the same species collected from different reef locations, and cultured under similar conditions, respond to varying treatments of temperature and nutrients in controlled laboratory-based experiments. We used populations of the LBF *Amphistegina lobifera*, which are found abundantly across the continental shelf on the Great Barrier Reef, and whose distribution is linked to thermal range and dissolved nitrate^23^. Each parameter was tested separately in two two-factor (reef site and the respective parameter) experiments. We investigated the threshold of the onset of oxidative stress and of disruptions in the calcification of this species by analysing bleaching frequency, survivorship and growth rates. We also performed a total antioxidant capacity assay and analysed the activity of Ca-ATPase. Specifically, we investigate if physiology differs between stable and variable environments, and if *A. lobifera* collected from different reefs can acclimate to changes in the environment over short time scales (up to 30 days). We hypothesised that *A. lobifera* populations living in relatively stable environments are more sensitive to shifts in environmental conditions than those living on reefs where physicochemical parameters are more variable.

## Results

### Temperature experiment

**Figure 1:**
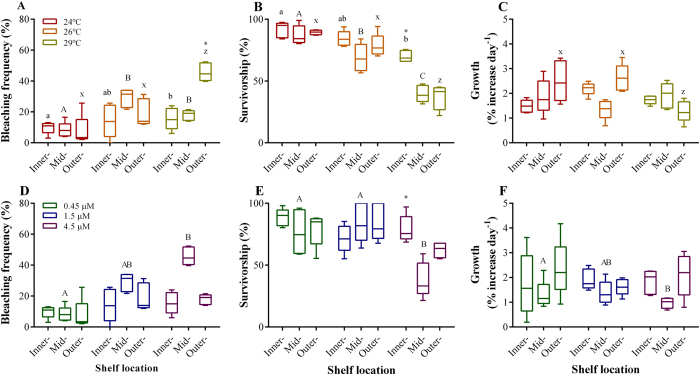
Bleaching frequency, survivorship and growth rates of *Amphistegina lobifera*. Individuals collected from different reef sites across the northern Great Barrier Reef where exposed to different conditions of temperature (**A?C**) and nitrate (**D?F**) after a 30-day period. Data are plotted as mean ± 95% C.I. (N = 5). Bars indicate minimum and maximum values. Different small and capital letters indicate statistically significant differences (Tukey’s HSD *posthoc* test; P < 0.05) in mean values among experimental groups of individuals collected from the same reef site. Asterisk (*) indicates significantly different mean values among reef sites within each experimental condition.

In the first experiment we investigated the effect of temperature on foraminifera from three different locations on a gradient from inshore to offshore. Bleaching frequency did not vary significantly between reef sites, and temperature levels also did not have a significant individual effect. However, the interaction of these two factors was significant (Table 1). Mean bleaching frequency in the outer-shelf population exposed to highest temperature level was above 40% (Fig. 1A), while at inner- and mid-shelf populations it remained below 30% (Tukey’s HSD *posthoc* test; Fig. 1A; Table 1; Supplementary Table S1 online).

Survivorship did not vary significantly among temperature conditions, but this response was site dependent (Table 1). The interaction between these factors was significant (Fig. 1B; Table 1). Foraminiferal populations collected from the mid- and outer-shelf sites presented survivorship percentages of ~40% and ~35%, respectively, at 29 °C by the end of the experiment. In contrast, populations from the inner-shelf reef region were significantly more resistant (Tukey’s HSD *posthoc* test, Fig. 1B; Supplementary Table S2 online), with high survivorship ( > 85%) even in the high temperature treatment. No significant mortality was observed for *A. lobifera* individuals exposed to ambient conditions across reef sites (overall mortality of ~10%).

The factors temperature or reef site had no significant effect on growth rates of *Amphistegina*, but the interaction between these factors was significant (Table 2). Elevated temperature had no effect on inner- and mid-shelf individuals. Growth in specimens collected from outer-shelf reefs was greater at 24 °C and 26 °C than for their inner- and mid-shelf reefs representatives. At these temperatures, outer-shelf reef individuals grew ~30% more than inner- and mid-shelf populations. Although not significant, it is noteworthy that growth rates of outer-shelf foraminifera at 29 °C were 33% and 20% lower than those from reefs located in the inner and mid shelves, respectively (Fig. 1C; Supplementary Table S3 online).

**Figure 2:**
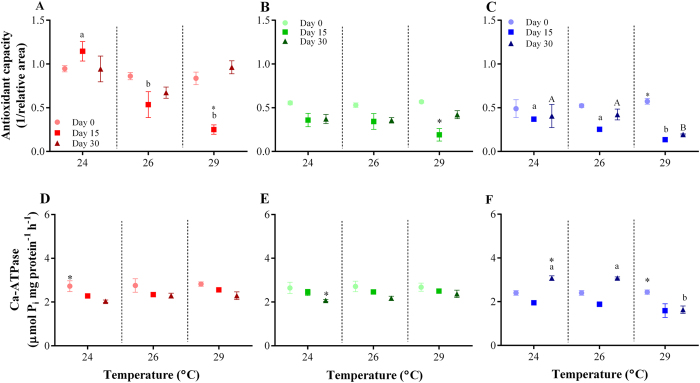
Elevated temperature effect on total antioxidant capacity (**A?C**) and Ca-ATPase (**D?F**) activity of *Amphistegina lobifera*. Individuals were collected from different reef sites (i.e., inner, mid and outer shelves) across the northern Great Barrier Reef and exposed to varying levels of temperature. Inner-shelf reefs: (**A,D**); Mid-shelf reef: (**B,E**); Outer-shelf reefs: (**C,F**). Data are plotted as mean ± SEM (N = 3). Different small and capital letters indicate statistically significant differences (Tukey’s HSD *posthoc* test; P < 0.05) in mean values among experimental groups of individuals collected from the same reef site. Asterisk (*) indicates significantly different mean values among reef sites within each experimental condition.

Reef site and elevated temperature had a significant effect on holobiont antioxidant capacity over the experimental period. The interaction of these factors (i.e., reef site x temperature levels) was also significant (Table 3). In general, initial antioxidant capacity of individuals collected from inner-shelf reefs was ~20% higher than that of foraminifera from the other reef sites (Tukey’s HSD *posthoc* test, Fig. 2A–C; Supplementary Table S4 online). At 24 °C a slight, but not significant, increase in antioxidant capacity was observed in inner-shelf *A. lobifera* population, a trend that was not observed in mid- and outer-shelf populations. At 26 °C all three populations responded similarly. *Amphistegina lobifera* collected from reef sites located at inner and mid shelves that were exposed to 26 and 29 °C were able to recover following a decline in antioxidant capacity, after 30 days (Tukey’s HSD *posthoc* test, Fig. 2A,B). Thermal stress was more pronounced in *A. lobifera* collected from outer-shelf reef sites, and antioxidant capacity in this population was ~70% lower than inner- and mid-shelf counterparts at 29 °C after 30 days (Tukey’s HSD *posthoc* test, Fig. 2C; Supplementary Table S4 online). Moreover, at 29 °C outer-shelf individuals were not capable of returning to their original antioxidant state after either 15 or 30 days (Tukey’s HSD *posthoc* test, Fig. 2C), as opposed to inner- and mid-shelf ones.

Reef site and temperature had a significant interactive effect on Ca-ATPase activity (Table 3). Individuals collected from reefs located in the inner and mid shelves showed a similar trend in Ca-ATPase activity, with a gradual reduction in activity over time for most temperature conditions observed, including ambient controls (Tukey’s HSD *posthoc* test, Fig. 2D–F; Table 2; Supplementary Table S5 online). In outer-shelf reef foraminifera, enzyme activity increased with time when exposed to 24 °C and 26 °C treatments, but was significantly inhibited at 29 °C (Tukey’s HSD *posthoc* test, Fig. 2F; Supplementary Table S5 online). This inhibition represented a decline of 32% from day 0 to day 30.

### Nutrient experiment

In the second experiment, we investigated the same responses of *A. lobifera* individuals collected from the same reef sites across a gradient inshore to offshore. Bleaching frequency differed significantly among reef sites, although it remained low (>25%) throughout the experiment; and increasing nitrate concentration did not have a significant effect among populations analysed (Tukey’s HSD *posthoc* test, Fig. 1D–F; Table 1). However, the interactive effect of reef site and elevated nitrate concentration in the water was significant (Table 1; Supplementary Table S6 online). Foraminifera exposed to control nitrate concentrations did not show any significant reef site effect in bleaching frequencies, and bleaching frequency was consistent across nitrate treatments in the inner-shelf populations. However, we detected significantly higher bleaching frequency in populations from the reef sites located in the mid and outer shelves exposed to 1.5 and 4.5 μM NO_3_^−^. Bleaching in the mid-shelf foraminifera increased progressively with addition of nitrate in the water, and at 4.5 μM NO_3_^−^ was as high as 46% (Tukey’s HSD *posthoc* test, Fig. 1D).

Survivorship did not vary among reef sites but was significantly different between treatments (Fig. 1E; Table 1). The interaction between reef sites and nitrate levels was also significant. A low survivorship percentage was observed for the *A. lobifera* population collected from the mid-shelf reef when exposed to the highest concentration of nitrate. At this concentration a mean of only ~40% of individuals survived after 30 days.

As with bleaching and survivorship, the interaction between different nitrate levels and reef site was significant on growth rates of *A. lobifera* populations (Table 2). However, increases of nitrate did not significantly affected populations from inner- and outer-shelf reef sites. Growth rates of foraminiferal populations that live in the mid-shelf reef were slightly reduced by elevated nitrate (Tukey’s HSD *posthoc* test, Fig. 1F), and were reduced by ~20% when compared to control treatments (i.e., 0.45 μM NO_3_^−^; Fig. 1F; Supplementary Table S8 online).

**Figure 3:**
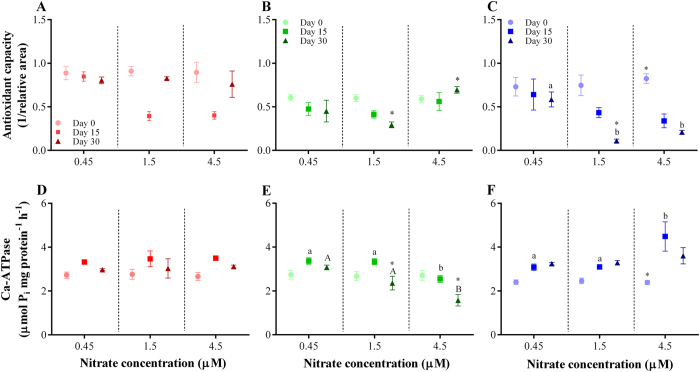
Effect of nitrification on total antioxidant capacity (**A?C**) and Ca-ATPase (**D?F**) activity of *Amphistegina lobifera*. Individuals were collected from different reef sites (i.e. inner-, mid- and outer-shelfs) across the northern Great Barrier Reef and exposed to varying levels of dissolved inorganic nitrate. Inner-shelf reefs: (**A,D**); Mid-shelf reef: (**B,E**); Outer-shelf reefs: (**C,F**). Data are plotted as mean ± SEM (N = 3). Different small and capital letters indicate statistically significant differences (Tukey’s HSD *posthoc* test; P < 0.05) in mean values among experimental groups of individuals collected from the same reef site. Asterisk (*) indicates significantly different mean values among reef sites within each experimental condition.

Addition of nitrate in the water had a significant effect on the antioxidant capacity response of populations from the reef sites studied, but the interaction of these two factors was not significant (Table 3). Antioxidant capacity of individuals fluctuated over the course of the experiment, and so the interaction between reef sites, nitrate concentration and time was significant (Table 3). In general, *A. lobifera* collected from inner-shelf reefs showed similar patterns of decrease following increase of antioxidant levels for the two higher nitrate concentrations tested (i.e., 1.5 and 4.5 μM). During the first 15 days antioxidant capacity dropped, but it recovered by the end of the experiment (Tukey’s HSD *posthoc* test, Fig. 3A–C; Supplementary Table S9 online). Conversely, outer-shelf foraminifera exposed to nutrient concentration above 1.5 μM NO_3_^−^ exhibited significant and continuous declines in antioxidant capacity after 15 and 30 days when compared to ambient conditions (Fig. 3C). Oxidative stress was most severe in outer-shelf individuals, where antioxidant capacity declined by 87% and 76% when exposed to 1.5 and 4.5 μM NO_3_^−^, respectively.

The individual effect of reef site was significant for the activity of Ca-ATPase of *A. lobifera*, whereas different concentrations of nitrate were not (Table 3). Individuals from different sites also presented significant different Ca-ATPase activity responses through time (Fig. 3D–F; Supplementary Table S10 online). Interaction of reef site, time and nitrate level was significant. Inner-shelf foraminifera showed similar values in Ca-ATPase activity after 15 and 30 days, with no significant differences between treatments or exposure time to increased concentrations of nitrate (Fig. 3D). However, mid-shelf foraminifera showed a gradual inhibition of enzyme activity with increased nitrate concentrations, which persisted through time (Tukey’s HSD *posthoc* test, Fig. 3E). At 4.5 μM NO_3_^−^, activity dropped to ~40% below that of control samples after 30 days. Outer-shelf samples showed a marginal, but not significant, increase in enzyme activity over time when exposed to 0.45 and 1.5 μM NO_3_^−^ (Fig. 3F). However, when exposed to 4.5 μM NO_3_^−^, activity significantly increased for the first 15 days, before stabilising by the experiment’s conclusion, and reached levels above those of lower nitrate levels (Tukey’s HSD *posthoc* test, Fig. 3F; Table S10).

## Discussion

This study revealed the effects of elevated temperature and dissolved nitrate on the biology and physiology of *Amphistegina lobifera* differed depending on their source location. Our study aimed at identifying populations of LBF that could potentially be acclimated/adapted to different environmental conditions. Our study populations collected from different reef sites along a cross-shelf gradient showed divergent survivorship, bleaching frequency, growth rates and physiological responses when exposed to various regimes of temperature and dissolved nitrate. Individuals collected from the reef sites located in the inner shelf are, in general, more resilient to increasing values of temperature and nitrate than foraminifera that live in reefs located in the mid- and outer-shelf regions, which were more sensitive to changes in parameters tested (Table 4), confirming our original hypothesis.

Bleaching frequency under elevated temperatures differed significantly between treatments and was reef site-dependent, and it generally increased with heat stress. Bleaching is usually regarded as a general stress response, driven by the breakdown of the symbiosis between malfunctioning algae and the host^24^. Talge and Hallock^17^ showed that in symbiont-bearing foraminifera bleaching can be triggered by exposure to temperature above their normal thermal range, but without mortality. It is possible that individuals capable of digesting their symbionts can avoid extensive cellular damage and death, showing the possible short-term protective effect of bleaching in foraminifera. Nonetheless, if bleaching persists, mortality will likely follow as Talge and Hallock^17^ found no evidence that bleached chambers could recover.

Our biochemical analyses showed that the antioxidant states of inner- and mid-shelf *A. lobifera* were able to recover to their pre-experimental levels (i.e., control levels) by the end of the experiment, demonstrating that individuals subjected to natural continuous fluctuations of physicochemical parameters may be able to acclimate to new conditions in as little as 30 days. However, individuals collected from outer shelf were not able to survive oxidative stress, as antioxidant levels declined significantly after 15 days and remained lower than control levels until the end of the experiment (Fig. 2C). Temperatures of 29 °C also caused impairment of Ca-ATPase activity in *A. lobifera* from the outer-shelf reefs, and consequently affected foraminiferal growth rates (Fig. 1C).

**Figure 4:**
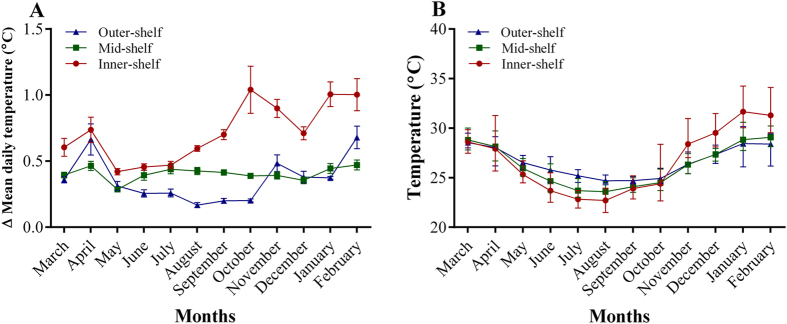
Temperature profiles of reefs located in the Far North region of the Great Barrier Reef, Australia. Daily (**A**) and seasonal (**B**) temperature profiles recorded at depths between 6 and 7.5 m on the reef slope of inner-, mid-, and outer-shelf reefs across the northern Great Barrier Reef, Australia. Inner-shelf reef: Cape Flattery; mid-shelf reef: Lizard Island; and outer-shelf reef: Yonge reef. Data are expressed as mean, maximum and minimum temperature values (GLZ; reef site × month; P < 0.001). Data available at: http://data.aims.gov.au.

Differences in biochemical responses observed among *A. lobifera* collected from different reef sites are likely related to daily and seasonal fluctuations of temperature that vary on a cross-shelf scale. At the reef site locations located in the outer-shelf region (Yonge and Day Reefs), average SSTs range between 24 °C in July (winter) and 28 °C in February (summer) (Fig. 4). However, at inner-shelf reefs such as Linnet Reef, daily fluctuations of temperature are more pronounced, even with increasing depth. Additionally, inner-shelf foraminifera experience transitory peaks of temperatures higher than 29 °C during summer (Fig. 4B), which can also be influenced by lagoonal outflow after low tides^25^, which is thought to explain their higher tolerance to temperatures above their optimal maxima of ~25 °C^17^. It is plausible that, similar to corals in the Florida Keys^26^, foraminifera from inshore reefs have the capacity to tolerate greater temperature ranges (i.e., higher differences between maximum and minimum temperatures) than those from offshore reefs, and are therefore more resilient to bleaching and mortality associated with thermal stress.

Similar to the temperature experiment, *A. lobifera* collected from Martin and Linnet Reefs were the most resistant to high concentrations of dissolved nitrate. Corals and other coral reef organisms can grow in areas that naturally experience elevated nutrient concentrations^27^. It is possible that organisms that live in regions with seasonal input of nutrients have an elevated tolerance threshold to increasing nitrate. On the GBR, reefs located in the inner-shelf are likely to experience elevated nutrients during seasonal runoff over the summer-wet season. Inshore chlorophyll *a* concentrations (indicative of eutrophication) are generally twice as high as on offshore reefs^27^,^28^. *In situ* measurements of dissolved nitrate concentration at Martin and Linnet Reefs commonly falls between 0.86 to 1.25 μM. Extreme events such as cyclones can increase nitrate concentration up to 1.85 μM, which is ~3-fold higher than concentrations observed in mid-shelf reefs around Lizard Island (personal observations). *Amphistegina* is capable of establishing symbioses with a range of different species of diatoms^29^. Thus, it is possible that *A. lobifera* collected from different reef sites along the cross-shelf gradient host diatoms with varying nutritional requirements. As a result, inner-shelf *A. lobifera* are able to thrive within a broader range of nitrate concentration (i.e., oligo- and mesotrophic waters) than mid- and outer-shelf populations, without suffering mortality.

Although we did observe marked survivorship in the outer-shelf population, nitrate concentrations above 1.5 μM induced a reduction in antioxidant capacity after 15 days of exposure (Fig. 3C). Conversely, these individuals were able to regulate intracellular functions that resulted in a stimulation of Ca-ATPase activity. As a result, growth rates were not directly affected by increased concentration of nitrate. This indicates that even individuals that live in areas with irregular nutrient inputs have developed mechanisms to survive these conditions, probably resulting from seasonal upwelling that might affect that reef region^30^. Upwelled water can contain up to 3 μM NO_3_^−^ at the shelf break, and nutrient concentrations during upwelling on the outer-shelf consistently exceed nutrient concentrations detected around inner- and mid-shelf reefs by an order of magnitude^31^. For example, *in situ* measured concentration of nitrate around Lizard Island falls between 0.08 and 0.45 μM, which is approximately 10 × lower than predictions for outer-shelf regions during upwelling. At Lizard Island, the natural nitrate levels are not only low, but also less variable, due to the distance from the mainland and lack of upwelling. It is possible that for this reason, mid-shelf *A. lobifera* individuals were most sensitive to increasing nitrate concentration.

At a population level, this variability in the range of responses observed could be linked to factors such as phenotypic plasticity or genetic variation among the reef sites we analysed. *Amphistegina lobifera* have the ability to produce distinct phenotypes when exposed to different environments^32^,^33^. Phenotypic plasticity allows organisms to adjust their physiology and morphology according to local conditions^34^, and provides the potential for organisms to respond rapidly and effectively to local environmental changes^35^. Additionally, Alve and Goldstein^36^ argued that shallow-water benthic foraminifera have poor dispersal mechanisms, and morphologically similar populations that occupy comparable environments in separate regions may be genetically distinct^36^,^37^,^38^. For example, environments that are thermally unstable among generations are predicted to favour genotypes that function within a wide range of temperature^39^. Therefore, foraminifera that live in the cooler and less variable thermal environment of the outer-shelf reefs are more sensitive to heat stress than inner- and mid-shelf individuals that experience higher daily and seasonal temperature fluctuations. Lastly, LBFs can host different algae species of the same type^6^. This suggests that the host could shuffle symbiont species according to environmental conditions^40^, as also observed for corals^41^. Therefore, it is plausible that the higher tolerance to changes in environmental conditions in *A. lobifera* collected from inner-shelf reefs could also be related to their type of diatom symbionts^42^.

In summary, we have shown that differential tolerance to elevated temperature and nitrate susceptibility in *A. lobifera* is likely to be related to their local habitat. Evidence of physiological plasticity can be inferred from variation in survivorship, bleaching frequency and enzymatic activity in *A. lobifera* exposed to varying conditions of elevated temperature and nitrification under lab-controlled conditions. Our study provides the fundamental analysis that is crucial to understanding the acclimation potential of holobiont organisms to changing environmental conditions. Studies on the interactive effect of these parameters are required to determine whether their combined effects are additive, antagonistic or synergistic, and to investigate how multiple stressors influence calcification and oxidative stress responses in holobiont organisms. Moreover, whether an adaptive component exists or if the transgenerational response is flexible has important implications for the response of local populations to the effects of environmental changes.

## Methods

### Study sites

The GBR is the world’s largest continuous coral reef ecosystem and is characterised by a continental cross-shelf gradient that results in dramatic ecosystem variation from the inshore coastal zones to the offshore outer-shelf refs^43^. This gradient is due to the exceptional width of the continental shelf in most areas, allowing for strong variations in factors such as depth, nutrient and sediment loads^43^. Variation of SST can be observed along and across the length of the GBR^43^,^44^. While cross-shelf patterns in mean SST can be consistent, daily and seasonal variability is substantially greater at inshore locations compared to mid- and outer-shelf areas^44^,^45^ (Fig. 4).

Coral reefs located along the Queensland coast are also impacted by freshwater runoff, carrying sediments and nutrients from the adjacent continental land mass^46^,^47^. Inner-shelf reefs located closer to the coast are more likely to be affected by fluctuating nutrient loads and turbidity^48^,^49^,^50^. For example, during the austral monsoon season concentrations of dissolved inorganic nitrogen in the inshore water column can be as high as 10 μM^51^. Nonetheless, outer-shelf reefs are sporadically influenced by nutrient-enriched water from the offshore thermocline (i.e., upwelling), and dissolved inorganic nitrate levels can reach values up to 3 μM^31^.

### Sample collection

**Figure 5:**
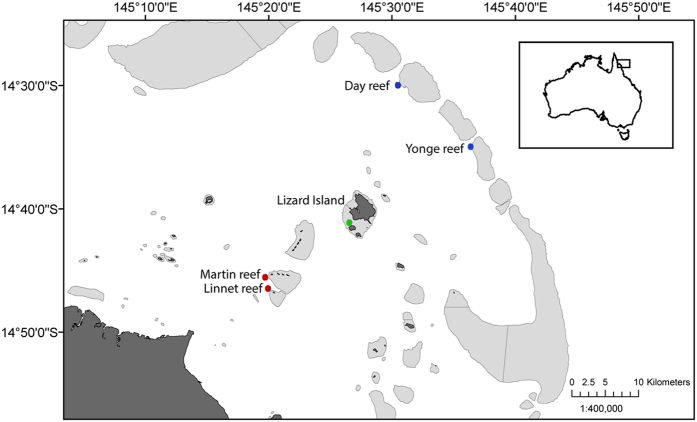
Location of sites. Sampling sites across the continental shelf on the Far North section of the Great Barrier Reef, Australia. Inner-shelf reefs: Martin and Linnet reefs; Mid-shelf reef: Lizard Island; Outer-shelf reefs: Yonge and Day reefs. Map was generated using the software ArcGIS v10.2 ( www.esri.com).

Dead coral rubble colonised by *A. lobifera* was collected from reef sites located in a temperature and nutrient gradient from inshore to offshore of the northern GBR in August and September 2013 (Fig. 5). Samples were collected by SCUBA divers from the leeward reef slopes located on: (1) the inner-shelf – Martin (14° 45.3′ S; 145° 20.1′ E) and Linnet (14° 46.7′ S; 145° 20.3′ E) reefs, (2) the mid-shelf – Lizard Island (14° 14.4′ S; 145° 27.9′ E), and (3) the outer-shelf – Yonge (14° 35.8′ S; 145° 37.4′ E) and Day reefs (14° 29.5′ S; 145° 30.9′ E). All samples were collected at depths of 6.0 to 9.5 m (corrected to lowest astronomical tide). Samples were amalgamated into three groups: inner-, mid- and outer-shelf individuals. Rubble was brought to the laboratory located at the Lizard Island Research Station and processed following the established sampling procedures^52^. Briefly, pieces of rubble were scrubbed using a toothbrush and resulting sediments were poured into Petri dishes for further separation of *A. lobifera* specimens. Adult *A. lobifera* ( > 0.7 mm in diameter) of uniform brown colour that displayed reticulopodial activity (indicative of viability) were selected and acclimated for five days prior to commencing experiments.

### Experiment design and setup

The effect of different temperature and nutrient regimes on the foraminifera was studied over a period of 30 days in flow-through (500 ml min^−1^) outdoor aquaria at the Lizard Island Research Station. For each two-factor experiment, three treatments were used with each treatment consisting of five independent replicates (tanks). Eighty specimens from each shelf location were haphazardly assigned and placed into Petri dishes in each replicate tank. Each Petri dish contained pieces of dead coral skeleton, so that individuals could attach themselves. Within each tank, half the individuals (40) were used for biomarker analyses and the other half (40) were used for survivorship and bleaching assessment. Twenty individuals were separated at the beginning of the experiment, and 20 individuals were sampled after 15 and 30 days. In addition, five individuals from each site were randomly selected from each of the 15 tanks to measure growth rates.

### Temperature experiment

Incoming unfiltered natural seawater was either fed straight into the tanks (ambient controls: 24 ± 0.2 °C = winter conditions) or stored in two different sumps, heated to either 26 ± 0.5 °C or 29 ± 0.3 °C using feedback controlled heaters and gravity fed into each relevant treatment tank. The temperature range was chosen based on the recorded seasonal range across the study locations, with a mean upper temperature limit of 29 °C during the summer and 24 °C during the winter at mid- and outer-shelf reefs (Fig. 4B). Temperature was monitored throughout the duration of the experiment using HOBO^®^ data loggers in each sump and in one of the replicate tanks per treatment. For the temperature experiments, outdoor aquaria were placed under shade cloth, reducing ambient light levels to 80.3 ± 5.6 μmol photon m^−2^ s^−1^ at midday. Natural nutrient levels were 0.44 ± 0.08 and 0.1 ± 0.007 μM of dissolved inorganic nitrate and phosphate, respectively.

### Nutrient experiment

Nutrient levels were manipulated by adding nitrate (NO_3_^−^) to the seawater. Natural dissolved inorganic phosphate concentrations were kept constant at 0.07 ± 0.005 μM. Similar to the temperature experiment, water was either fed straight into the tanks (ambient control: 0.45 ± 0.05 μM NO_3_^−^) or stored in sumps. Two solutions of NaNO_3_^−^ at 0.2 mM and 0.8 mM were added into each sump at a constant rate (1 ml min^−1^) using peristaltic pumps. The final nitrate treatments consisted of 1.55 ± 0.09 and 4.51 ± 0.04 μM NO_3_^−^, which were fed into the respective tanks at 500 ml min^−1^. Nitrate concentrations were chosen based on the natural cross-shelf range, and the highest concentration utilised was based on peaks of nitrate input previously reported for the inshore studied area^51^. Nitrate concentration was monitored daily using a Nitrate Pro test kit (RedSea^©^) and two filtered (0.45 μm) water samples were taken from each tank once a week for further laboratory analysis. Natural irradiance was reduced to 81.5 ± 1.6 μmol photon m^−2^ s^−1^ using shade cloth, and water temperature was kept at 24 ± 0.8 °C throughout the 30-day experiment.

### Survivorship and bleaching frequency

We started each experiment with 40 individuals per replicate. To determine the frequency of bleaching, we counted the number of individuals that showed any sign of symbiont loss (ranging from small white spots to extensive white or “mottled” areas) and determined the percentage of bleached individuals following Hallock *et al.*^52^. Likewise, the number of dead individuals (empty shells without reticulopodial activity) was assessed after 30 days and survivorship was calculated as percentages. Individuals that underwent asexual reproduction, as evidenced by empty shell surrounded by newborn offspring, were not included in the total number of dead individuals.

### Growth rate

Surface area was analysed by digital photographs taken of the cross-sectional shell surface area throughout the experiment^20^,^53^. Digital photographs (Microscope SteREO Discovery V8 with attached AxioCam Arc 5s, Zeiss, Australia) of each of the five assigned individuals per site per tank were taken at the beginning and at the end of each experiment. Photographs were analysed using the software ImageJ^54^ to automatically trace the surface area of the specimens and calculate area gain (derived from pixel area gain) over the course of the experiment. Growth rates of *A. lobifera* were based on overall means of each Petri dish as the shells of this species do not possess characteristic differences that would allow the tracking of individual specimens. Growth rate (% surface area day^−1^) was determined using the equations of ter Kuile and Erez^33^ as follows:


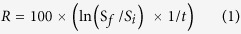


Where, S_*f*_ and S_*i*_ are the final and initial surface area size, respectively, *t* is the time in days and *R* is the growth rate in %. Average initial surface area of analysed specimens did not deviate between shelf locations for each experiment (*Temperature*: One-way ANOVA, F_(2, 42)_ = 1.91; *P* = 0.16; *Nutrient*: One-way ANOVA, F_(2, 42)_ = 3.03; *P* = 0.08).

### Determination of total antioxidant capacity

The total antioxidant capacity (TAC) assay was performed to measure the biological resistance to various kinds of oxyradicals in order to predict their adverse effects on the physiological condition of the holobionts. TAC was measured using the fluorescence technique following the protocol described in Amado *et al.*^55^ and modified by Prazeres *et al.*^56^. Each sample was homogenized in a Tris-HCl (100 mM) buffer containing EDTA (2 mM) and MgCl_2_ (5 mM) and protein concentration was adjusted to 0.75 mg protein ml^−1^ using the Quant-iT Protein Assay (Invitrogen, USA). We added 10 μl of supernatant from each sample to a black 96-well microplate together with 127.5 μl of reaction buffer. For the reaction, 7.5 μl of 2,2′-Azobis (2-methylpropionitrile) (1 mM) was further added to each sample. Finally, the fluorescent probe 2′,7′ dichlorofluoresceindiacetate (H_2_DCF-DA) was added to all wells at a final concentration of 40 μM. Fluorescence was read (excitation: 488 ηm; emission: 525 ηm) every 5 min in a microplate reader (Synergy2 – BioTek) for up to 45 min at 37 °C. Results were expressed as the inverse of the relative area and calculated according to Amado *et al.*^55^.

### Activity of Ca-ATPase

Ca^2+^-ATPase is a membrane-bound enzyme that is suggested to play an important role in calcium homeostasis and calcification^57^,^58^. Ca-ATPase activity of *A. lobifera* holobionts was determined according to Prazeres *et al.*^58^. Briefly, samples were homogenized in a buffer solution containing 500 mM sucrose, 150 mM KCl, 20 mM Tris Base, 1 mM _DL_-dithiothreitol, and 0.1 mM phenylmethylsulfonyl, adjusted to pH 7.6. Homogenates were then centrifuged and the resulting supernatant was adjusted to a concentration of 0.1 mg of protein ml^−1^ using the Quant-iT Protein Assay (Thermo Fisher Scientific, Australia). Ca-ATPase was quantified by incubating samples with 100 μl of working buffer at 30 °C for 30 min. The reaction was started by the addition of 3 mM ATP and stopped by placing the samples on ice for 10 min. Samples were analysed following the method of Fiske and Subbarow^59^, and activity was calculated according to Prazeres *et al.*^58^.

### Data analyses

Each experiment was analysed independently. Bleaching frequency and survivorship were analysed with a Generalised Linear Mixed Model (GLMM) using the package *lme4* in R^60^. As they represent percentages, we used a binomial GLMM instead of an arc sine transformed Analysis of Variance (ANOVA)^61^. GLMM was carried out to determine differences between reef sites for each environmental parameter (i.e., temperature or nitrate). Reef site and environmental parameter for each experiment were used as fixed factors. Significance of single and interaction effects of fixed factors was quantified using Type III Sum of Squares ANOVA. A factor detailing each ‘reef site x treatment’ combination was employed for a Tukey’s HSD *posthoc* test, using the package *multcomp*. R scripts for the analysis can be found in the online Supplementary Material. Growth rate was analysed using a Two-way ANOVA with Type III Sum of Squares with reef site and environmental parameter as fixed factors. In this case, data was logit transformed prior to the ANOVA^61^. Total antioxidant capacity and Ca-ATPase activity were analysed using a Two-way Repeated Measures ANOVA (rmANOVA), and reef site, environmental parameter and time (i.e., 0, 15 and 30 days) were used as fixed factors. Prior to performing rmANOVA, the assumption for sphericity was tested using Mauchley’s test. When indicated, rmANOVA was followed by Tukey’s HSD test. All data were checked for homogeneity of variance as well as normality prior to ANOVA analyses using Bartlett’s and Shapiro-Wilk’s tests, respectively. In cases where ANOVA assumptions were violated, data were log (*x* + 1) transformed. Data of daily temperature variation, shown in Fig. 4, for each shelf location over the time period from March 2006 to February 2007 were compared using a Generalised Linear Model (GLZ) with a Poisson error distribution and log link function. GLZ was chosen as the data violated parametric assumptions even after transformations. In this case, shelf location and month was used as fixed factors. In all cases, the significance level adopted was 95% (α = 0.05). These analyses were conducted using the statistical software Statistica 12^62^.

## Additional Information

**How to cite this article**: Prazeres, M. *et al.* Influence of local habitat on the physiological responses of large benthic foraminifera to temperature and nutrient stress. *Sci. Rep.* **6**, 21936; doi: 10.1038/srep21936 (2016).

## Supplementary Material

Supplementary Information

## Figures and Tables

**Table 1:** Generalised Linear Mixed model results for bleaching frequency and survivorship of *Amphistegina lobifera* populations collected from different reef sites and exposed to varying conditions of temperature and nitrate.

Parameter tested	Response variable	Source	*df*	χ^2^	P value
Temperature	Bleaching frequency	Site	2	5.02	0.08
		Temperature	2	1.04	0.59
		Site*Temperature	4	43.17	**<0.001**
	Survivorship	Site	2	66.39	**<0.001**
		Temperature	2	4.79	0.09
		Site*Temperature	4	27.07	**<0.001**
Nitrate	Bleaching frequency	Site	2	6.48	**0.04**
		Nitrate	2	1.02	0.60
		Site*Nitrate	4	44.07	**<0.001**
	Survivorship	Site	2	12.15	**0.002**
		Nitrate	2	21.26	**<0.001**
		Site*Nitrate	4	131.35	**<0.001**

Significant values (P < 0.05) are in bold.

**Table 2:** Two-way Analysis of Variance results for growth rates of *Amphistegina lobifera* populations collected from different reef sites and exposed to varying conditions of temperature and nitrate.

Parameter tested	Source	*df*	MS	F	P value
Temperature	Site	2	0.02	1.63	0.21
	Temperature	2	0.02	2.13	0.13
	Site*Temperature	4	0.11	6.68	**<0.01**
	Residuals	36	0.02		
Nitrate	Site	2	0.03	2.79	0.08
	Nitrate	2	0.06	6.20	**<0.01**
	Site*Nitrate	4	0.04	4.71	**<0.01**
	Residuals	36	0.01		

Significant values (P < 0.05) are in bold.

**Table 3:** Repeated Measures ANOVA results of biomarkers analysis (i.e. antioxidant capacity assay and Ca-ATPase activity) for *Amphistegina lobifera* individuals collected from inner-, mid- and outer-shelf reefs exposed to varying conditions of temperature and nitrate.

Parameter tested	Response variable	Source	*df*	MS	F	P value
Temperature	Antioxidant capacity	Site	1	0.08	158.90	**<0.001**
		Treatment	2	0.01	28.26	**<0.001**
		Time	2	0.05	62.60	**<0.001**
		Site*Treatment	4	0.004	8.60	**<0.001**
		Site*Treatment*Time	8	0.005	6.39	**<0.001**
		Residual	36	0.007		
	Ca-ATPase	Site	1	0.46	5.88	**0.01**
		Treatment	2	0.54	6.88	**<0.01**
		Time	2	1.51	19.55	**<0.001**
		Site*Treatment	4	0.32	4.01	**0.02**
		Site*Treatment*Time	8	0.31	4.10	**<0.01**
		Residual	36	0.07		
Nitrate	Antioxidant capacity	Site	1	0.54	21.00	**<0.01**
		Treatment	2	0.18	7.19	**<0.01**
		Time	2	0.59	32.97	**<0.01**
		Site*Treatment	4	0.07	2.73	0.06
		Site*Treatment*Time	8	0.05	3.10	**<0.01**
		Residual	36	0.02		
	Ca-ATPase	Site	1	2.12	7.81	**<0.01**
		Treatment	2	0.48	1.80	0.19
		Time	2	1.06	6.02	**<0.01**
		Site*Treatment	4	0.91	3.34	**0.03**
		Site*Treatment*Time	8	0.62	3.56	**<0.01**
		Residual	36	0.17		

Significant values (P < 0.05) are in bold.

**Table 4:**
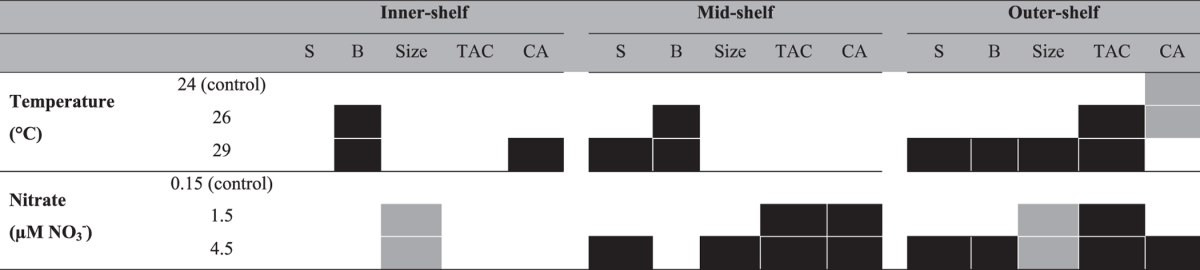
Results summary for variables measured in *Amphistegina lobifera* exposed to different conditions of temperature and nitrate after 30 days.

Grey and black boxes indicate a significant positive or negative effect, respectively (*P* < 0.05), while white boxes indicate no significant effect for variables analysed. S: Survivorship; B: Bleaching frequency; TAC: Total antioxidant capacity; and CA: Ca-ATPase activity.
