# Supplementary material for: Influence of local habitat on the physiological responses of large benthic foraminifera to temperature and nutrient stress
Source: Sci Rep. 2016 Feb 23;6:21936. doi: 10.1038/srep21936 (PMC4763287; doi:10.1038/srep21936)
Supplement: Supplementary Information [file srep21936-s1.pdf]

Supplementary information

Influence of local habitat on the physiological responses of large benthic foraminifera to temperature and nutrient stress

Martina Prazeres[1,*], Sven Uthicke[2], and John M. Pandolfi[1]

[1]Australian Research Council (ARC) Centre of Excellence for Coral Reef Studies and School of Biological Sciences, The University of Queensland, Brisbane, Queensland, 4072, Australia

[2]Australian Institute of Marine Science, PMB No 3, Townsville, Queensland 4810, Australia

*Corresponding author

Current address: Comparative Genomics Centre, College of Public Health, Medical and Veterinary Sciences, James Cook University, Townsville, Queensland, 4811, Australia

Phone: +61 7 4781 5395

Email: martina.defreitasprazeres@jcu.edu.au

R scripts

Survivorship and bleaching frequency analyses:

GLMM

```
library(lme4)
M1<-glm(cbind(V1,V2)~ Treatment*Site,family=binomial,data=data file)
summary(M1)
```

ANOVA

```
library(car)
Anova(M1,type="III")
```

Tukey's HSD *post hoc* test

```
library(multcomp)
M.tukeyS<-glmer(cbind(V1,V2)~Treatment*Site+(1|Tank),family=binomial,data=data file)
summary(glht(M.tukeyS,linfct=mcp(Treatment*Site="Tukey")))
```

Table S1. Tukey's HSD *post hoc* test of the pairwise comparisons of bleaching frequency among populations collected from inner-, mid- and outer-shelf reefs exposed to different *temperature* conditions.
The bold numbers indicated significant pairwise comparisons (P < 0.05).

Bleaching frequency – TEMPERATURE		Inner-shelf 24°C	Inner-shelf 26°C	Inner-shelf 29°C	Mid-shelf 24°C	Mid-shelf 26°C	Mid-shelf 29°C	Outer-shelf 24°C	Outer-shelf 26°C	Outer-shelf 29°C
Inner-shelf	24°C									
Inner-shelf	26°C	**<0.01**								
Inner-shelf	29°C	**<0.01**	0.52							
Mid-shelf	24°C	0.07	**<0.01**	**<0.01**						
Mid-shelf	26°C	**<0.01**	0.16	**<0.01**	**<0.01**					
Mid-shelf	29°C	**<0.01**	0.75	**<0.01**	**<0.01**	1.00				
Outer-shelf	24°C	0.08	**<0.01**	**<0.01**	**<0.01**	**<0.01**	**<0.01**			
Outer	26°C	**<0.01**	**<0.01**	**<0.01**	**<0.01**	0.26	0.72	0.48		
Outer	29°C	**<0.01**	1.00	**0.04**	**<0.01**	0.69	0.19	**<0.01**	**0.02**	

Table S2. Tukey's HSD *post hoc* test of the pairwise comparisons of survivorship among populations collected from inner-, mid- and outer-shelf reefs exposed to different *temperature* conditions.
The bold numbers indicated significant pairwise comparisons (P < 0.05).

Survivorship – TEMPERATURE		Inner-shelf 24°C	Inner-shelf 26°C	Inner-shelf 29°C	Mid-shelf 24°C	Mid-shelf 26°C	Mid-shelf 29°C	Outer-shelf 24°C	Outer-shelf 26°C	Outer-shelf 29°C
Inner-shelf	24°C									
Inner-shelf	26°C	0.09								
Inner-shelf	29°C	**<0.01**	<0.01							
Mid-shelf	24°C	0.40	0.98	**<0.01**						
Mid-shelf	26°C	**<0.01**	**<0.01**	0.99	**<0.01**					
Mid-shelf	29°C	**<0.01**	**<0.01**	**<0.01**	**<0.01**	**<0.01**				
Outer-shelf	24°C	0.97	0.59	**<0.01**	0.97	**<0.01**	**<0.01**			
Outer	26°C	**<0.01**	0.46	0.37	0.15	**0.03**	**<0.01**	**<0.01**		
Outer	29°C	**<0.01**	**<0.01**	**<0.01**	**<0.01**	**<0.01**	0.99	**<0.01**	**<0.01**	

Table S3. Tukey's HSD *post hoc* test of the pairwise comparisons of growth rates among populations collected from inner-, mid- and outer-shelf reefs exposed to different *temperature* conditions.
The bold numbers indicated significant pairwise comparisons (P < 0.05).

Growth rates – TEMPERATURE		Inner-shelf 24°C	Inner-shelf 26°C	Inner-shelf 29°C	Mid-shelf 24°C	Mid-shelf 26°C	Mid-shelf 29°C	Outer-shelf 24°C	Outer-shelf 26°C	Outer-shelf 29°C
Inner-shelf	24°C									
Inner-shelf	26°C	0.43								
Inner-shelf	29°C	0.99	0.93							
Mid-shelf	24°C	0.98	0.96	1.00						
Mid-shelf	26°C	1.00	0.12	0.79	0.72					
Mid-shelf	29°C	0.91	0.99	1.00	1.00	0.53				
Outer-shelf	24°C	0.18	1.00	0.68	0.76	**0.04**	0.90			
Outer	26°C	0.07	0.99	0.41	0.49	**0.01**	0.68	1.00		
Outer	29°C	0.98	0.06	0.57	0.49	1.00	0.32	**0.02**	**<0.01**	

Table S4. Tukey's HSD *post hoc* test of the pairwise comparisons of antioxidant capacity among populations collected from inner-, mid- and outer-shelf reefs exposed to different *temperature* conditions at 0, 15 and 30 days of experiment.

The bold numbers indicated significant pairwise comparisons ($P < 0.05$).

Antioxidant capacity – TEMPERATURE			Inner-shelf 24°C 0	Inner-shelf 24°C 15	Inner-shelf 24°C 30	Inner-shelf 26°C 0	Inner-shelf 26°C 15	Inner-shelf 26°C 30	Inner-shelf 29°C 0	Inner-shelf 29°C 15	Inner-shelf 29°C 30	Mid-shelf 24°C 0	Mid-shelf 24°C 15	Mid-shelf 24°C 30	Mid-shelf 26°C 0	Mid-shelf 26°C 15	Mid-shelf 26°C 30	Mid-shelf 29°C 0	Mid-shelf 29°C 15	Mid-shelf 29°C 30	Outer-shelf 24°C 0	Outer-shelf 24°C 15	Outer-shelf 24°C 30	Outer-shelf 26°C 0	Outer-shelf 26°C 15	Outer-shelf 26°C 30	Outer-shelf 29°C 0	Outer-shelf 29°C 15	Outer-shelf 29°C 30
Inner-shelf	24°C	0																											
Inner-shelf	24°C	15	0.99																										
Inner-shelf	24°C	30	1.00	0.99																									
Inner-shelf	26°C	0	1.00	0.82	1.00																								
Inner-shelf	26°C	15	**0.00**	**0.00**	**0.00**	**0.02**																							
Inner-shelf	26°C	30	0.67	**0.01**	0.65	0.96	0.72																						
Inner-shelf	29°C	0	1.00	0.68	1.00	1.00	**0.01**	0.98																					
Inner-shelf	29°C	15	**0.00**	**0.00**	**0.00**	**0.00**	0.28	**0.00**	**0.00**																				
Inner-shelf	29°C	30	1.00	1.00	1.00	1.00	**0.00**	0.27	1.00	**0.00**																			
Mid-shelf	24°C	0	**0.04**	**0.00**	**0.04**	0.15	1.00	1.00	0.23	**0.01**	**0.01**																		
Mid-shelf	24°C	15	**0.00**	**0.00**	**0.00**	**0.00**	0.96	**0.01**	**0.00**	1.00	**0.00**	0.37																	
Mid-shelf	24°C	30	**0.00**	**0.00**	**0.00**	**0.00**	1.00	**0.04**	**0.00**	0.98	**0.00**	0.78	1.00																
Mid-shelf	26°C	0	**0.02**	**0.00**	**0.01**	0.06	1.00	0.99	0.11	**0.03**	**0.00**	1.00	0.46	0.89															
Mid-shelf	26°C	15	**0.00**	**0.00**	**0.00**	**0.00**	0.98	**0.01**	**0.00**	1.00	**0.00**	0.37	1.00	1.00	0.69														
Mid-shelf	26°C	30	**0.00**	**0.00**	**0.00**	**0.00**	1.00	**0.02**	**0.00**	1.00	**0.00**	0.56	1.00	1.00	0.85	1.00													
Mid-shelf	29°C	0	0.06	**0.00**	0.06	0.21	1.00	1.00	0.32	**0.01**	**0.01**	1.00	0.19	0.59	1.00	0.28	0.44												
Mid-shelf	29°C	15	**0.00**	**0.00**	**0.00**	**0.00**	**0.02**	**0.00**	**0.00**	1.00	**0.00**	**0.00**	0.89	0.47	**0.00**	0.80	0.62	**0.00**											
Mid-shelf	29°C	30	**0.00**	**0.00**	**0.00**	**0.00**	1.00	0.24	**0.00**	0.69	**0.00**	0.98	1.00	1.00	1.00	1.00	1.00	0.97	0.20										
Outer-shelf	24°C	0	**0.00**	**0.00**	**0.00**	**0.02**	1.00	0.91	**0.04**	0.10	**0.00**	1.00	0.74	0.98	1.00	0.85	0.95	1.00	**0.01**	1.00									
Outer-shelf	24°C	15	**0.00**	**0.00**	**0.00**	**0.00**	1.00	**0.04**	**0.00**	0.98	**0.00**	0.70	1.00	1.00	0.89	1.00	1.00	0.58	0.48	1.00	0.99								
Outer-shelf	24°C	30	**0.02**	**0.00**	**0.02**	0.09	1.00	1.00	0.15	**0.02**	**0.00**	1.00	0.38	0.83	1.00	0.51	0.70	1.00	**0.00**	1.00	1.00	0.88							
Outer-shelf	26°C	0	**0.04**	**0.00**	**0.04**	0.16	1.00	1.00	0.25	**0.01**	**0.01**	1.00	0.24	0.67	1.00	0.34	0.52	1.00	**0.00**	0.98	1.00	0.67	1.00						
Outer-shelf	26°C	15	**0.00**	**0.00**	**0.00**	**0.00**	0.10	**0.00**	**0.00**	1.00	**0.00**	**0.00**	1.00	0.83	**0.01**	0.98	0.92	**0.00**	1.00	0.36	**0.03**	0.83	**0.01**	**0.01**					
Outer-shelf	26°C	30	**0.01**	**0.00**	**0.01**	**0.04**	1.00	0.96	0.06	0.06	**0.00**	1.00	0.62	0.96	1.00	0.74	0.89	1.00	**0.00**	1.00	1.00	0.96	1.00	1.00	**0.04**				
Outer-shelf	29°C	0	0.07	**0.00**	0.06	0.23	1.00	1.00	0.35	**0.01**	**0.01**	1.00	0.17	0.55	1.00	0.24	0.40	1.00	**0.00**	0.94	1.00	0.54	1.00	1.00	**0.00**	1.00			
Outer-shelf	29°C	15	**0.00**	**0.00**	**0.00**	**0.00**	**0.00**	**0.00**	**0.00**	0.97	**0.00**	**0.00**	0.33	0.08	**0.00**	0.23	0.13	**0.00**	1.00	**0.01**	**0.00**	0.08	**0.00**	**0.00**	1.00	**0.00**	**0.00**		
Outer-shelf	29°C	30	**0.00**	**0.00**	**0.00**	**0.00**	**0.03**	**0.00**	**0.00**	1.00	**0.00**	**0.00**	0.94	0.57	**0.00**	0.87	0.71	**0.00**	1.00	0.16	**0.01**	0.57	**0.00**	**0.00**	1.00	**0.00**	**0.00**	1.00	

Table S5. Tukey's HSD *post hoc* test of the pairwise comparisons of Ca-ATPase activity among populations collected from inner-, mid- and outer-shelf reefs exposed to different *temperature* conditions at 0, 15 and 30 days of experiment.

The bold numbers indicated significant pairwise comparisons ($P < 0.05$).

Ca-ATPase – TEMPERATURE			Inner-shelf<br>24°C<br>0	Inner-shelf<br>24°C<br>15	Inner-shelf<br>24°C<br>30	Inner-shelf<br>26°C<br>0	Inner-shelf<br>26°C<br>15	Inner-shelf<br>26°C<br>30	Inner-shelf<br>29°C<br>0	Inner-shelf<br>29°C<br>15	Inner-shelf<br>29°C<br>30	Mid-shelf<br>24°C<br>0	Mid-shelf<br>24°C<br>15	Mid-shelf<br>24°C<br>30	Mid-shelf<br>26°C<br>0	Mid-shelf<br>26°C<br>15	Mid-shelf<br>26°C<br>30	Mid-shelf<br>29°C<br>0	Mid-shelf<br>29°C<br>15	Mid-shelf<br>29°C<br>30	Outer-shelf<br>24°C<br>0	Outer-shelf<br>24°C<br>15	Outer-shelf<br>24°C<br>30	Outer-shelf<br>26°C<br>0	Outer-shelf<br>26°C<br>15	Outer-shelf<br>26°C<br>30	Outer-shelf<br>29°C<br>0	Outer-shelf<br>29°C<br>15	Outer-shelf<br>29°C<br>30
Inner-shelf	24°C	0																											
Inner-shelf	24°C	15	0.29																										
Inner-shelf	24°C	30	1.00	**0.01**																									
Inner-shelf	26°C	0	1.00	0.11	1.00																								
Inner-shelf	26°C	15	0.19	1.00	**0.00**	0.08																							
Inner-shelf	26°C	30	1.00	0.19	1.00	1.00	0.13																						
Inner-shelf	29°C	0	1.00	0.42	1.00	1.00	0.30	1.00																					
Inner-shelf	29°C	15	0.97	1.00	0.22	0.82	1.00	0.92	0.99																				
Inner-shelf	29°C	30	0.88	1.00	0.12	0.63	1.00	0.78	0.95	1.00																			
Mid-shelf	24°C	0	1.00	0.59	0.98	1.00	0.45	1.00	1.00	1.00	0.99																		
Mid-shelf	24°C	15	0.77	1.00	0.07	0.47	1.00	0.63	0.89	1.00	1.00	0.95																	
Mid-shelf	24°C	30	1.00	0.98	0.59	0.99	0.95	1.00	1.00	1.00	1.00	1.00	1.00																
Mid-shelf	26°C	0	1.00	0.97	0.65	0.99	0.92	1.00	1.00	1.00	1.00	1.00	1.00	1.00															
Mid-shelf	26°C	15	0.82	1.00	0.08	0.53	1.00	0.70	0.92	1.00	1.00	0.98	1.00	1.00	1.00														
Mid-shelf	26°C	30	0.11	1.00	**0.00**	**0.03**	1.00	0.06	0.18	0.99	1.00	0.29	1.00	0.85	0.79	1.00													
Mid-shelf	29°C	0	1.00	0.99	0.55	0.98	0.96	1.00	1.00	1.00	1.00	1.00	1.00	1.00	1.00	1.00	0.88												
Mid-shelf	29°C	15	0.91	1.00	0.13	0.67	1.00	0.81	0.97	1.00	1.00	0.99	1.00	1.00	1.00	1.00	1.00	1.00											
Mid-shelf	29°C	30	0.15	1.00	**0.00**	0.05	1.00	0.09	0.24	1.00	1.00	0.38	1.00	0.91	0.88	1.00	1.00	0.93	1.00										
Outer-shelf	24°C	0	1.00	0.14	1.00	1.00	0.09	1.00	1.00	0.87	0.70	1.00	0.55	1.00	1.00	0.61	**0.05**	0.99	0.74	0.07									
Outer-shelf	24°C	15	**0.02**	1.00	**0.00**	**0.01**	1.00	**0.01**	**0.04**	0.86	0.96	0.08	0.99	0.48	0.42	0.98	1.00	0.52	0.95	1.00	**0.01**								
Outer-shelf	24°C	30	1.00	0.13	1.00	1.00	0.08	1.00	1.00	0.85	0.67	1.00	0.52	0.99	1.00	0.58	**0.04**	0.99	0.71	0.06	1.00	**0.01**							
Outer-shelf	26°C	0	1.00	0.23	1.00	1.00	0.15	1.00	1.00	0.95	0.84	1.00	0.70	1.00	1.00	0.76	0.08	1.00	0.86	0.12	1.00	**0.02**	1.00						
Outer-shelf	26°C	15	1.00	0.60	0.98	1.00	0.46	1.00	1.00	1.00	0.99	1.00	0.96	1.00	1.00	0.98	0.30	1.00	0.99	0.39	1.00	0.09	1.00	1.00					
Outer-shelf	26°C	30	1.00	**0.01**	1.00	1.00	**0.00**	1.00	1.00	0.22	0.12	0.98	0.07	0.59	0.65	0.08	**0.00**	0.55	0.13	**0.00**	1.00	**0.00**	1.00	1.00	0.98				
Outer-shelf	29°C	0	1.00	0.24	1.00	1.00	0.16	1.00	1.00	0.95	0.84	1.00	0.71	1.00	1.00	0.77	0.09	1.00	0.87	0.12	1.00	**0.02**	1.00	1.00	1.00	1.00			
Outer-shelf	29°C	15	1.00	0.82	0.90	1.00	0.70	1.00	1.00	1.00	1.00	1.00	1.00	1.00	1.00	1.00	0.52	1.00	1.00	0.62	1.00	0.19	1.00	1.00	1.00	0.90	1.00		
Outer-shelf	29°C	30	**0.00**	0.08	**0.00**	**0.00**	0.14	**0.00**	**0.00**	**0.00**	**0.01**	**0.00**	**0.01**	**0.00**	**0.00**	**0.01**	0.24	**0.00**	**0.00**	0.17	**0.00**	0.60	**0.00**	**0.00**	**0.00**	**0.00**	**0.00**	**0.00**	

Table S6. Tukey's HSD *post hoc* test of the pairwise comparisons of bleaching frequency among populations collected from inner-, mid- and outer-shelf reefs exposed to different *nitrate* conditions.

The bold numbers indicated significant pairwise comparisons (P < 0.05).

Bleaching frequency – NITRATE	Inner-shelf 0.45 µM	Inner-shelf 1.5 µM	Inner-shelf 4.5 µM	Mid-shelf 0.45 µM	Mid-shelf 1.5 µM	Mid-shelf 4.5 µM	Outer-shelf 0.45 µM	Outer-shelf 1.5 µM	Outer-shelf 4.5 µM
Inner-shelf 0.45 µM									
Inner-shelf 1.5 µM	0.75								
Inner-shelf 4.5 µM	0.40	0.99							
Mid-shelf 0.45 µM	0.99	0.25	0.07						
Mid-shelf 1.5 µM	**<0.01**	**<0.01**	**<0.01**	**<0.01**					
Mid-shelf 4.5 µM	**<0.01**	**<0.01**	**<0.01**	**<0.01**	**<0.01**				
Outer-shelf 0.45 µM	0.99	0.25	0.07	1.00	**<0.01**	**<0.01**			
Outer-shelf 1.5 µM	**0.02**	0.53	0.95	**<0.01**	**0.04**	**<0.01**	**<0.01**		
Outer-shelf 4.5 µM	0.08	0.94	0.99	**<0.01**	0.05	**<0.01**	**<0.01**	0.99	

Table S7. Tukey's HSD *post hoc* test of the pairwise comparisons of survivorship among populations collected from inner-, mid- and outer-shelf reefs exposed to different *nitrate* conditions.

The bold numbers indicated significant pairwise comparisons (P < 0.05).

Survivorship – NITRATE	Inner-shelf 0.45 µM	Inner-shelf 1.5 µM	Inner-shelf 4.5 µM	Mid-shelf 0.45 µM	Mid-shelf 1.5 µM	Mid-shelf 4.5 µM	Outer-shelf 0.45 µM	Outer-shelf 1.5 µM	Outer-shelf 4.5 µM
Inner-shelf 0.45 µM									
Inner-shelf 1.5 µM	**0.01**								
Inner-shelf 4.5 µM	0.26	0.94							
Mid-shelf 0.45 µM	**<0.01**	0.98	1.00						
Mid-shelf 1.5 µM	0.92	**<0.01**	0.96	0.90					
Mid-shelf 4.5 µM	**<0.01**	**<0.01**	**<0.01**	**<0.01**	**<0.01**				
Outer-shelf 0.45 µM	**<0.01**	0.89	1.00	0.99	0.98	**<0.01**			
Outer-shelf 1.5 µM	0.90	**<0.01**	0.97	0.92	1.00	**<0.01**	0.99		
Outer-shelf 4.5 µM	**<0.01**	0.83	**<0.01**	0.22	**<0.01**	**<0.01**	0.09	**<0.01**	

Table S8. Tukey's HSD *post hoc* test of the pairwise comparisons of growth rates among populations collected from inner-, mid- and outer-shelf reefs exposed to different *nitrate* conditions.

The bold numbers indicated significant pairwise comparisons (P < 0.05).

Growth rates – NITRATE	Inner-shelf 0.45 µM	Inner-shelf 1.5 µM	Inner-shelf 4.5 µM	Mid-shelf 0.45 µM	Mid-shelf 1.5 µM	Mid-shelf 4.5 µM	Outer-shelf 0.45 µM	Outer-shelf 1.5 µM	Outer-shelf 4.5 µM
Inner-shelf 0.45 µM									
Inner-shelf 1.5 µM	0.23								
Inner-shelf 4.5 µM	0.08	1.00							
Mid-shelf 0.45 µM	0.23	1.00	1.00						
Mid-shelf 1.5 µM	0.27	1.00	1.00	1.00					
Mid-shelf 4.5 µM	1.00	0.54	0.26	0.53	0.61				
Outer-shelf 0.45 µM	1.00	0.20	0.07	0.19	0.24	1.00			
Outer-shelf 1.5 µM	0.12	1.00	1.00	1.00	1.00	0.34	0.10		
Outer-shelf 4.5 µM	1.00	**0.04**	**0.01**	**0.04**	0.06	**0.03**	1.00	**0.02**	

Table S9. Tukey's HSD *post hoc* test of the pairwise comparisons of antioxidant capacity among populations collected from inner-, mid- and outer-shelf reefs exposed to different *nitrate* conditions at 0, 15 and 30 days of experiment.

The bold numbers indicated significant pairwise comparisons ($P < 0.05$).

Antioxidant capacity – NITRATE			Inner-shelf 0.45 µM 0	Inner-shelf 0.45 µM 15	Inner-shelf 0.45 µM 30	Inner-shelf 1.5 µM 0	Inner-shelf 1.5 µM 15	Inner-shelf 1.5 µM 30	Inner-shelf 4.5 µM 0	Inner-shelf 4.5 µM 15	Inner-shelf 4.5 µM 30	Mid-shelf 0.45 µM 0	Mid-shelf 0.45 µM 15	Mid-shelf 0.45 µM 30	Mid-shelf 1.5 µM 0	Mid-shelf 1.5 µM 15	Mid-shelf 1.5 µM 30	Mid-shelf 4.5 µM 0	Mid-shelf 4.5 µM 15	Mid-shelf 4.5 µM 30	Outer-shelf 0.45 µM 0	Outer-shelf 0.45 µM 15	Outer-shelf 0.45 µM 30	Outer-shelf 1.5 µM 0	Outer-shelf 1.5 µM 15	Outer-shelf 1.5 µM 30	Outer-shelf 4.5 µM 0	Outer-shelf 4.5 µM 15	Outer-shelf 4.5 µM 30
Inner-shelf	0.45 µM	0																											
Inner-shelf	0.45 µM	15	0.91																										
Inner-shelf	0.45 µM	30	0.70	1.00																									
Inner-shelf	1.5 µM	0	1.00	1.00	1.00																								
Inner-shelf	1.5 µM	15	**0.00**	0.06	0.14	**0.01**																							
Inner-shelf	1.5 µM	30	0.90	1.00	1.00	1.00	0.06																						
Inner-shelf	4.5 µM	0	1.00	1.00	1.00	1.00	**0.02**	1.00																					
Inner-shelf	4.5 µM	15	**0.00**	0.07	0.16	**0.02**	1.00	0.10	**0.01**																				
Inner-shelf	4.5 µM	30	0.56	1.00	1.00	1.00	0.31	1.00	1.00	0.25																			
Mid-shelf	0.45 µM	0	**0.03**	0.93	0.99	0.66	0.99	0.97	0.74	0.99	1.00																		
Mid-shelf	0.45 µM	15	**0.00**	0.27	0.51	0.09	1.00	0.38	0.11	1.00	0.77	1.00																	
Mid-shelf	0.45 µM	30	**0.00**	0.18	0.37	0.05	1.00	0.26	0.07	1.00	0.63	1.00	1.00																
Mid-shelf	1.5 µM	0	**0.03**	0.92	0.99	0.63	0.99	0.97	0.71	0.99	1.00	1.00	1.00	1.00															
Mid-shelf	1.5 µM	15	**0.00**	0.08	0.20	**0.02**	1.00	0.13	**0.03**	1.00	0.40	1.00	1.00	1.00	0.99														
Mid-shelf	1.5 µM	30	**0.00**	**0.00**	**0.01**	**0.00**	1.00	**0.01**	**0.00**	1.00	**0.04**	0.61	1.00	1.00	0.52	1.00													
Mid-shelf	4.5 µM	0	**0.02**	0.89	0.98	0.57	0.99	0.95	0.65	1.00	1.00	1.00	1.00	1.00	1.00	1.00	0.70												
Mid-shelf	4.5 µM	15	**0.01**	0.76	0.93	0.40	1.00	0.86	0.48	1.00	0.99	1.00	1.00	1.00	1.00	1.00	0.85	1.00											
Mid-shelf	4.5 µM	30	0.22	1.00	1.00	0.98	0.68	1.00	0.99	0.73	1.00	1.00	0.98	0.93	1.00	0.78	0.16	1.00	1.00										
Outer-shelf	0.45 µM	0	0.39	1.00	1.00	1.00	0.46	1.00	1.00	0.51	1.00	1.00	0.90	0.79	1.00	0.57	0.08	1.00	1.00	1.00									
Outer-shelf	0.45 µM	15	0.08	0.99	1.00	0.85	0.92	1.00	0.90	0.94	1.00	1.00	1.00	1.00	1.00	0.96	0.40	1.00	1.00	1.00	1.00								
Outer-shelf	0.45 µM	30	**0.02**	0.87	0.98	0.54	1.00	0.94	0.62	1.00	1.00	1.00	1.00	1.00	1.00	1.00	0.73	1.00	1.00	1.00	1.00	1.00							
Outer-shelf	1.5 µM	0	0.48	1.00	1.00	1.00	0.38	1.00	1.00	0.42	1.00	1.00	0.83	0.71	1.00	0.48	0.06	1.00	1.00	1.00	1.00	1.00	1.00						
Outer-shelf	1.5 µM	15	**0.00**	0.13	0.28	**0.03**	1.00	0.19	**0.05**	1.00	0.53	1.00	1.00	1.00	1.00	1.00	1.00	1.00	1.00	0.88	0.70	0.99	1.00	0.48					
Outer-shelf	1.5 µM	30	**0.00**	**0.00**	**0.00**	**0.00**	0.77	**0.00**	**0.00**	0.73	**0.00**	**0.02**	0.31	0.44	**0.02**	0.67	1.00	**0.03**	0.06	**0.00**	**0.00**	**0.01**	**0.03**	**0.00**	0.42				
Outer-shelf	4.5 µM	0	0.89	1.00	1.00	1.00	0.09	1.00	1.00	0.11	1.00	0.98	0.39	0.27	0.97	0.14	**0.01**	0.95	0.87	1.00	1.00	1.00	0.94	1.00	0.20	**0.00**			
Outer-shelf	4.5 µM	15	**0.00**	0.16	0.33	**0.04**	1.00	0.23	0.06	1.00	0.59	1.00	1.00	1.00	1.00	1.00	1.00	1.00	1.00	0.91	0.76	0.99	1.00	0.67	1.00	0.48	0.16		
Outer-shelf	4.5 µM	30	**0.00**	**0.00**	**0.00**	**0.00**	1.00	**0.00**	**0.00**	1.00	**0.01**	0.18	0.86	0.94	0.20	0.99	1.00	0.24	0.38	**0.03**	**0.01**	0.09	0.26	**0.01**	0.97	1.00	**0.00**	0.90	

Table S10. Tukey's HSD *post hoc* test of the pairwise comparisons of Ca-ATPase among populations collected from inner-, mid- and outer-shelf reefs exposed to different *nitrate* conditions at 0, 15 and 30 days of experiment.

The bold numbers indicated significant pairwise comparisons ($P < 0.05$).

Ca-ATPase – NITRATE			Inner-shelf 0.45 µM 0	Inner-shelf 0.45 µM 15	Inner-shelf 0.45 µM 30	Inner-shelf 1.5 µM 0	Inner-shelf 1.5 µM 15	Inner-shelf 1.5 µM 30	Inner-shelf 4.5 µM 0	Inner-shelf 4.5 µM 15	Inner-shelf 4.5 µM 30	Mid-shelf 0.45 µM 0	Mid-shelf 0.45 µM 15	Mid-shelf 0.45 µM 30	Mid-shelf 1.5 µM 0	Mid-shelf 1.5 µM 15	Mid-shelf 1.5 µM 30	Mid-shelf 4.5 µM 0	Mid-shelf 4.5 µM 15	Mid-shelf 4.5 µM 30	Outer-shelf 0.45 µM 0	Outer-shelf 0.45 µM 15	Outer-shelf 0.45 µM 30	Outer-shelf 1.5 µM 0	Outer-shelf 1.5 µM 15	Outer-shelf 1.5 µM 30	Outer-shelf 4.5 µM 0	Outer-shelf 4.5 µM 15	Outer-shelf 4.5 µM 30
Inner-shelf	0.45 µM	0																											
Inner-shelf	0.45 µM	15	1.00																										
Inner-shelf	0.45 µM	30	1.00	1.00																									
Inner-shelf	1.5 µM	0	1.00	0.99	0.93																								
Inner-shelf	1.5 µM	15	1.00	1.00	1.00	1.00																							
Inner-shelf	1.5 µM	30	1.00	1.00	0.99	1.00	1.00																						
Inner-shelf	4.5 µM	0	0.97	0.47	0.30	1.00	0.70	1.00																					
Inner-shelf	4.5 µM	15	1.00	1.00	1.00	0.94	1.00	0.99	0.21																				
Inner-shelf	4.5 µM	30	1.00	1.00	1.00	1.00	1.00	1.00	0.99	0.99																			
Mid-shelf	0.45 µM	0	1.00	0.85	0.69	1.00	0.96	1.00	1.00	0.71	1.00																		
Mid-shelf	0.45 µM	15	1.00	1.00	1.00	1.00	1.00	1.00	0.86	1.00	1.00	0.98																	
Mid-shelf	0.45 µM	30	1.00	1.00	0.99	1.00	1.00	1.00	1.00	1.00	1.00	1.00	1.00																
Mid-shelf	1.5 µM	0	1.00	0.76	0.58	1.00	0.92	1.00	1.00	0.60	1.00	1.00	0.98	1.00															
Mid-shelf	1.5 µM	15	1.00	1.00	1.00	1.00	1.00	1.00	0.90	1.00	1.00	1.00	1.00	1.00	0.97														
Mid-shelf	1.5 µM	30	0.83	0.22	0.12	1.00	0.39	0.98	1.00	0.13	0.95	1.00	0.58	0.97	1.00	0.49													
Mid-shelf	4.5 µM	0	1.00	0.94	0.83	1.00	0.99	1.00	1.00	0.84	1.00	1.00	1.00	1.00	1.00	1.00	1.00												
Mid-shelf	4.5 µM	15	0.98	0.52	0.34	1.00	0.74	1.00	1.00	0.36	1.00	1.00	0.89	1.00	1.00	0.92	1.00	1.00											
Mid-shelf	4.5 µM	30	**0.00**	**0.00**	**0.00**	**0.04**	**0.00**	**0.02**	0.44	**0.00**	**0.01**	0.15	**0.00**	**0.01**	0.21	**0.00**	0.75	**0.05**	0.27										
Outer-shelf	0.45 µM	0	1.00	1.00	1.00	1.00	1.00	1.00	0.97	1.00	1.00	1.00	1.00	1.00	1.00	1.00	0.80	1.00	0.98	**0.00**									
Outer-shelf	0.45 µM	15	0.39	**0.04**	**0.02**	0.90	0.10	0.75	1.00	**0.02**	0.59	0.99	0.19	0.67	1.00	0.23	1.00	0.97	1.00	0.98	0.24								
Outer-shelf	0.45 µM	30	1.00	1.00	1.00	1.00	1.00	1.00	0.64	1.00	1.00	0.94	1.00	1.00	0.88	1.00	0.34	0.98	0.69	**0.00**	1.00	**0.05**							
Outer-shelf	1.5 µM	0	0.94	0.36	0.22	1.00	0.58	1.00	1.00	0.23	0.99	1.00	0.76	1.00	1.00	0.82	1.00	1.00	1.00	0.56	0.92	1.00	0.52						
Outer-shelf	1.5 µM	15	1.00	1.00	1.00	1.00	1.00	1.00	1.00	1.00	1.00	1.00	1.00	1.00	1.00	1.00	0.96	1.00	1.00	**0.01**	1.00	0.62	1.00	0.97					
Outer-shelf	1.5 µM	30	1.00	1.00	1.00	1.00	1.00	1.00	0.69	1.00	1.00	0.96	1.00	1.00	0.91	1.00	0.39	0.99	0.74	**0.00**	1.00	0.10	1.00	0.42	1.00				
Outer-shelf	4.5 µM	0	0.99	0.53	0.35	1.00	0.75	1.00	1.00	0.37	1.00	1.00	0.89	1.00	1.00	0.93	1.00	1.00	1.00	0.39	0.98	1.00	0.70	1.00	1.00	0.75			
Outer-shelf	4.5 µM	15	1.00	1.00	1.00	1.00	1.00	1.00	0.89	1.00	1.00	1.00	1.00	1.00	0.99	1.00	0.62	1.00	0.91	**0.00**	1.00	0.21	1.00	0.80	1.00	1.00	0.83		
Outer-shelf	4.5 µM	30	0.70	1.00	1.00	0.19	0.97	0.34	**0.01**	1.00	0.49	0.06	0.91	0.41	**0.04**	0.87	**0.00**	0.10	**0.01**	**0.00**	0.73	**0.00**	0.99	**0.01**	0.46	0.98	**0.01**	0.77	
